# Advanced Metrics and Early Predictors of Cardiogenic Shock

**DOI:** 10.7759/cureus.48100

**Published:** 2023-11-01

**Authors:** Nisarg Shah, Gabriella Orta, Sonia Daryanani, Kayvan Amini, Marc M Kesselman

**Affiliations:** 1 Cardiology, Nova Southeastern University Dr. Kiran C. Patel College of Osteopathic Medicine, Davie, USA; 2 Internal Medicine, Nova Southeastern University Dr. Kiran C. Patel College of Osteopathic Medicine, Davie, USA; 3 Rheumatology, Nova Southeastern University Dr. Kiran C. Patel College of Osteopathic Medicine, Davie, USA

**Keywords:** score, prognosis, clinical presentation, early predictors, cardiogenic shock

## Abstract

Cardiogenic shock (CS) can be defined as a range of illnesses that describes a state where cardiac output, which is the blood volume ejected from the left ventricle every minute that leads to tissue perfusion of nutrients, is insufficient in providing adequate oxygenation to the systemic circulation. The incidence of CS on admission has increased in recent years, with the six- and 12-month mortality rates remaining unchanged. The purpose of this literature review is to bring forth several parameters that have been utilized in the past 10 years to predict CS mortality. Further studies to implement these parameters in management algorithms, along with early screenings and advanced treatment options such as mechanical cardiac assist devices, can improve the mortality associated with CS. This literature-based review was conducted by evaluating current research focusing on the clinical presentation of CS and predictors of CS. PubMed served as the primary database for article retrieval due to access. Two searches were conducted: One for clinical presentations and advanced metrics for CS and another for early predictors for CS. Thirteen articles regarding clinical presentation and seven articles regarding early predictors were selected. Three tools/scores and five laboratory tests were identified that allowed clinicians to prognosticate outcomes in patients suffering from CS based on clinical and laboratory presentations. Examining these predictors will allow earlier intervention in the development of CS and potentially help lower the mortality rate of CS. The eventual creation of a scoring system that incorporates multiple metrics discussed in this review can provide the most accurate prognosis of CS, which can lead to more targeted interventions.

## Introduction and background

Cardiogenic shock (CS) can be defined as a range of illnesses that describes a state where cardiac output, which is the blood volume ejected from the left ventricle every minute that leads to tissue perfusion of nutrients, is insufficient in providing adequate oxygenation to the systemic circulation. This results in the eventual failure of organ systems within the body. One of the most commonly used clinical definitions was established by the Should We Emergently Revascularize Occluded Coronaries for Cardiogenic Shock trial and defines CS as systolic blood pressure (SBP) of less than 90 mmHg for greater than 30 minutes or the need for mechanical or pharmaceutical support to maintain a SBP greater than 90 mmHg. Other clinical markers used for the diagnosis of CS using other trials and guidelines are discussed (Table 1).

**Table 1:** Clinical criteria for the diagnosis of cardiogenic shock Reference: [1] SBP: Systolic blood pressure; EOD: End organ damage; HR: Heart rate; PCWP: Pulmonary capillary wedge pressure; MAP: Mean arterial pressure; AMS: Altered mental status; SHOCK: Should We Emergently Revascularize Occluded Coronaries for Cardiogenic Shock; IABP SOAP II: Intra-Aortic Balloon Pump in Cardiogenic Shock II; EHS-PCI = Euro Heart Survey Percutaneous Coronary Intervention Registry; ESC-HF: European Society of Cardiology Heart Failure; KAMIR-NIH: Korean Acute Myocardial Infarction Registry - National Institute of Health

Trial name	Criteria	Associated clinical presentation
SHOCK trial (1999)	SBP < 90 mmHg for >30 min or vasopressor support to maintain SBP > 90 mmHg Hemodynamics: Cardiac Index < 2.2 and PCWP > 15 mmHg	Evidence of EOD (Urine Output < 30 mL/hr, cool extremities, HR >= 60)
IABP SOAP II (2012)	MAP < 70 mmHg or SBP < 100 mmHg despite fluid resuscitation (at least 1 L of crystalloids or 500 mL of colloids)	Evidence of EOD (Altered Mental Status, mottled skin, Urine output < 0.5 mL/kg for one hour or serum lactate > 2 mmol/L)
EHS-PCI (2012)	SBP < 90 mmHg for 30 min or inotropes used to maintain SBP > 90 mmHg	Evidence of EOD and increased filling pressures
ESC-HF Guidelines (2016)	SBP < 90 mmHg with fluid resuscitation with clinical and lab evidence of EOD	Clinical: cold extremities, oliguria, AMS, narrow pulse pressure Lab: Metabolic acidosis, elevated serum lactate, and creatinine
Culprit-SHOCK (2017)	SBP < 90 mmHg for at least 30 min or infusion of catecholamines to maintain SBP > 90 mmHg	Impaired organ perfusion Clinical signs of pulmonary congestion, AMS, cold and clammy skin/limbs, urine output < 30 mL/hr, arterial lactate > 2 mmol/L
KAMIR-NIH (2018)	SBP < 90 mmHg for > 30 min or supportive intervention needed to maintain SBP > 90 mmHg	Evidence of EOD (AMS, urine output < 30 mL/hr, cold extremities)

Between 2007 and 2017, 383,983 patients in the United States presented with CS, and the hospital survival rate among them was 40.2% [2]. The incidence of CS on admission has increased in recent years, with the six- and 12-month mortality rates remaining unchanged at around 50% over the past 20 years [2-3]. A probable cause of this increase in incidence on admission is the improvement in healthcare networks, which has allowed effective transportation of patients within 90 minutes to hospitals equipped with a catheterization lab where percutaneous coronary intervention (PCI) can be performed [3]. As obesity, diabetes, and other diseases increase cardiovascular disease incidences, it is more paramount than ever to understand the development of CS so that it can be detected sooner [4-5]. Earlier interventions, such as metabolic screenings for biological markers and radiological imaging, can help spot several developing diseases, such as cardiomyopathy and valvular heart disease, which could be addressed prior to the development of CS [6]. The purpose of this literature review is to bring forth several parameters that have been utilized in the past 10 years to predict CS mortality. Further studies to implement these parameters in management algorithms, along with early screenings and advanced treatment options such as mechanical cardiac assist devices, can improve the mortality associated with CS.

Demographics

There are a number of factors that put certain patient demographics at an increased risk for CS versus others. Gaining insight into these factors among different ethnicities and backgrounds can help to target treatment more effectively and improve outcomes for patients with CS. In one study, researchers evaluated 157,892 patients who suffered from CS between 2003 and 2010. The investigators found that the incidence of CS was higher in patients older than 75 years of age as compared to patients younger than 75 years of age (9.4% vs. 7.3%, respectively). In addition, CS was higher among females as compared to their male counterparts (8.5% vs. 7.6%, respectively), and higher in Asians/Pacific Islanders than other races (11.4% vs 8% in whites, 6.9% in African Americans, and 8.6% in Hispanics) [7].

Patients aged 75 or older appear to have a higher risk of CS because of increased frailty, reduced functional reserve, a higher incidence of prior coronary interventions, and multiple comorbidities such as hypertension and type 2 diabetes mellitus [8]. In addition, women appear to be at a higher risk because they are less likely to develop ventricular dilatation during remodeling than men, which leads to deleterious short-term effects including an increased risk for CS. In addition, hypertension is also more common among women as compared to their male counterparts after 55 years of age and women have higher total cholesterol after the fifth decade of life. Estrogen (specifically 17-B-estradiol) is shown to aid in limiting cardiac apoptosis and mediating ischemia-reperfusion injury to the heart by reducing infarct size, weaning of estrogen after menopause is hypothesized to cause an increased risk in CS [9].

A higher risk of CS has been demonstrated among the Asian/Pacific Islander population, more specifically the South Asian (SA) population (including Indian, Pakistani, Bangladeshi, Nepali, and Sri Lankan descent). Due to differing socioeconomic statuses, access to healthcare services, education, and healthcare behaviors among other factors, the risk and outcomes of atherosclerotic cardiovascular disease can vary between SAs living in the United States and SAs living in their native countries [10]. In the Mediators of Atherosclerosis in South Asians Living in America (MASALA) study, Drs. Alka Kanaya and Namratha Kandula evaluated 1164 SA women and men aged 40-84. The investigators found that SAs living in the United States are more likely to die from heart disease than the general population [11]. SAs have increased visceral fat in the liver, abdominal area, and pericardium. Additionally, they have higher-grade coronary artery stenoses and a higher prevalence of multi-vessel disease. They also have impaired glucose tolerance leading to two-fold higher rates of type 2 diabetes mellitus as compared to their Caucasian counterparts [11].

Etiologies

CS etiologies can be divided into three main categories: Myogenic, mechanical, or arrhythmogenic. Myogenic causes of CS arise from pathologies associated with the myocardium and the muscle fibers of the heart itself that make it difficult to properly eject blood. This includes left and right ventricular myocardial infarctions (MI), where necrotic tissue is the result of cardiac ischemia, adenosine triphosphate (ATP) depletion, and calcium accumulation [12]. ATP serves as a crucial component of actin and myosin bridging for muscular contractions and decreased ATP availability leads to decreased myocardial contractions. MI progress in phases. Hypoxic damage induces the inflammatory phase in the first four days, where there is cardiac myocyte death and neutrophil infiltration. After cytokines, like transforming growth factor beta (TGF-β) are secreted to stimulate proliferation, the proliferative phase begins with angiogenesis (proliferation of blood vessels), collagen synthesis, myofibroblast differentiation, and cardiac myocyte regeneration for about three to four weeks. Once the proliferative phase subsides, the healing phase begins for about 4-6 weeks, marked by myofibroblast apoptosis and scar formation [13].

Cardiomyopathies, such as dilated or restrictive cardiomyopathies, are other primary muscle etiologies for deteriorating myocardial function [6]. Dilated cardiomyopathy is defined by having a left ventricular ejection fraction (LVEF) less than 45% and a left ventricular end-diastolic diameter greater than 117% of the predicted value after correcting for age and body surface area (normal values for left ventricular end-diastolic diameter in men and women are 42 to 59 mm and 39 to 54 mm, respectively) [14-15]. Restrictive cardiomyopathy is associated with a noncompliant left ventricle with dysfunctional diastolic filling, leading to high filling pressures [16]. Three of the most common types of restricted cardiomyopathies are cardiac amyloidosis, cardiac sarcoidosis, and cardiac hemochromatosis. Cardiac amyloidosis occurs with the deposition of amyloid protein in the heart, causing cellular toxicity, cardiomyocyte separation, and tissue stiffness. Cardiac sarcoidosis is an inflammatory disease that exposes the myocardium to T lymphocytes and noncaseating granulomas. Cardiac hemochromatosis is caused when excess dietary iron is absorbed and accumulated in the heart, leading to its dysfunction [17]. In all three of these conditions, the dilatory ability of the ventricular myocardium is compromised.

Mechanical reasons for CS are associated with valvular pathologies, such as mitral stenosis, mitral regurgitation, and aortic regurgitation, which prevent the necessary cardiac output from occurring, leading to eventual CS. Thrombi, such as a pulmonary embolism or a dislodged deep vein thrombosis, can obstruct the flow of blood and not allow the heart to output the mechanical force needed to adequately circulate the blood both within the heart and throughout the rest of the body.

Arrhythmogenic causes chronic bradycardia and atrial and ventricular arrhythmias and disrupts the speed and unidirectional flow of blood through the heart [6]. If electrical signals are not passed sequentially through the heart, sections will depolarize at uncoordinated times, which will create turbulent flow. Unidirectional flow is required to maintain cardiac output. Other etiologies of CS are discussed (Table 2).

**Table 2:** Common etiologies of cardiogenic shock Reference: [6] MI: Myocardial infarctions

Muscular Etiologies	Structural Etiologies	Rhythmic Etiologies
Right and Left ventricular MI Cardiomyopathy (dilated, restrictive, ischemic); Myocarditis Intoxication (alcohol, cocaine); Cytotoxic agents Drugs (calcium antagonists, Beta-blockers, antiarrhythmic, digitalis, antidepressants); Ventricular hypertrophy	Valvular heart disease (stenosis, regurgitation); Mechanical complications of MI Hypertrophic cardiomyopathy (obstructive, nonobstructive); Outflow tract obstruction by atrial or ventricular thrombi and heart tumors Cardiac or extracardiac filling disorders (pericardial tamponade, tension pneumothorax, pulmonary embolism); Aortic dissection Traumatic cardiac injury	Bradycardia Supraventricular or ventricular arrhythmias; Microbiological causes

CS can manifest in patients with or without acute coronary syndrome (ACS). ACS is an umbrella term used to refer to conditions where there is sudden, reduced blood flow to the heart. According to Sciaccaluga et al., about 60% to 80% of patients presenting with CS also have experienced ACS. The most common cause of CS is ST-elevation myocardial infarctions (STEMI) [18]. Meanwhile, patients presenting with nonACS-related CS suffer from a wide range of causes including hypertension, coronary artery disease, diabetes, cardiac arrhythmias, valvular disease, myocarditis, heart failure, and stress-induced cardiomyopathy [18-19]. Among patients who suffer from MI, left ventricular failure associated with an anterior MI appeared as the most frequent cause of CS. An anterior MI is an occlusion of the left anterior descending artery that supplies the anterior interventricular septum and anterior portion of the left ventricle. An inferior MI, which usually results from occlusion of the right coronary artery (or the left circumflex artery in left-dominant individuals), led to CS less frequently and was mostly due to mitral valve regurgitation, cardiac rupture with tamponade, or ventricular septal rupture [20]. The right coronary artery supplies the sinoatrial node, atrioventricular node, right ventricle, and right atrium.

Pathophysiology 

The progression of CS commonly involves a reduction in myocardial contractility primarily occurs, which in turn causes hypotension, systemic vasoconstriction, cardiac ischemia, and a decrease in cardiac output. Of significance is the peripheral vasoconstriction that occurs from inadequate circulatory compensation (when the vital organs, such as the heart and brain, do not get adequate oxygen), the peripheral vasculature vasoconstrictors to shunt blood to these vital organs. Shunting blood for an extended period results in eventual end-organ damage [1]. This vasoconstriction initially improves coronary perfusion by shunting peripheral blood flow to the coronary arteries and increasing the blood flow the cardiac tissues receive, but it ultimately increases cardiac afterload, which is the amount of work the heart must exert to send blood systemically. This leads to an increased cardiac inotropic requirement to get the same volume of blood to the systemic circulation, and increases the oxygen requirements of the heart, leading to further hypoxic conditions, additional damage, and a lack of viable contractile myocardium [1].

As the global heart muscle becomes ischemic and the right ventricle begins failing, the residual heart muscle has a reduced ability to adapt to various diastolic and systolic volumes and pressure needs. Right ventricular failure leads to right ventricle dilatation, which shifts the interventricular septum to the left, further decreasing left ventricle chamber size which in turn decreases LV filling and exacerbates the systemic hypoperfusion already occurring [1].

Systemic vasoconstriction also leads to the mobilization of blood from the splanchnic areas to compensate for the reduced preload, which is the sarcomere stretch within the cardiac myocytes at the end of diastole, due to the increased afterload. Reduced renal perfusion will cause the activation of the renin-angiotensin-aldosterone system to increase renal retention of fluids through the secretion of aldosterone to combat the perceived hypotension.

Upon restoration of blood pressure, through alternating vasoconstriction and vasodilation along with changes in vascular permeability to ischemic areas, an inflammation-like reaction termed reperfusion injury will occur, which leads to the activation of inflammatory mediators, such as interleukin-6 (IL-6), leukocytes, and tumor necrosis factor alpha (TNF-alpha), which will cause pathological vasodilation [6]. This vasodilation contributes to the mortality associated with CS as it propagates the sequence of events described above and creates a regressing cycle of the body constantly attempting to compensate for heightened requirements of function [1-4]. Vasodilation will also release nitric oxide synthase and peroxynitrite, which have cardiotoxic contractility effects. Peroxynitrite is formed from nitric oxide reacting with superoxide radicals. Peroxynitrite is a strong oxidant that causes DNA damage as well as inflammation activation that can lead to fibrosis and cell death of cardiac tissue [21]. The vasodilation caused by excessive nitric oxide results in barrier disruption through the vascular endothelium, which can potentially lead to the translocation of bacteria, leading to septic shock secondary to CS [6].

Diagnosis 

The diagnosis of CS involves an evaluation of clinical signs and symptoms, along with several hemodynamic measurements. Several clinical trials have been conducted, which have resulted in updated definitions and guidelines for diagnosing CS. A common guideline as a result of these trials is having an SBP less than 90 mmHg for greater than 30 minutes or the requirement of vasopressor support, which is the use of a medication that causes vasoconstriction and thus, an increase in mean arterial pressure, to maintain an SBP greater than 90 mmHg. A few diagnostic criteria also include signs of end organ damage (EOD), such as having a decreased urine output of less than 30 mL per hour (normal urine output is 800-2000 mL per day with an intake of around 2 L of fluids) and having cold extremities due to decreased blood circulation [6]. A comprehensive list of diagnostic criteria based on various trials and guidelines is listed in Table 1 [1].

Methods 

This literature-based review was conducted by evaluating current research focusing on the advanced metrics of CS and predictors of CS. PubMed served as the primary database for article retrieval due to open access. Further studies should incorporate additional databases covering additional languages to gain a more encompassing review. Two searches were conducted: one for clinical presentations and advanced metrics for CS and another for early predictors for CS. Both searches were conducted for the purpose of understanding the trends in clinical presentations of CS, which contributed to the reasoning behind why certain metrics were used to predict CS outcomes. Articles were first screened to ensure they were published in the English language after the year 2012.

**Figure 1:**
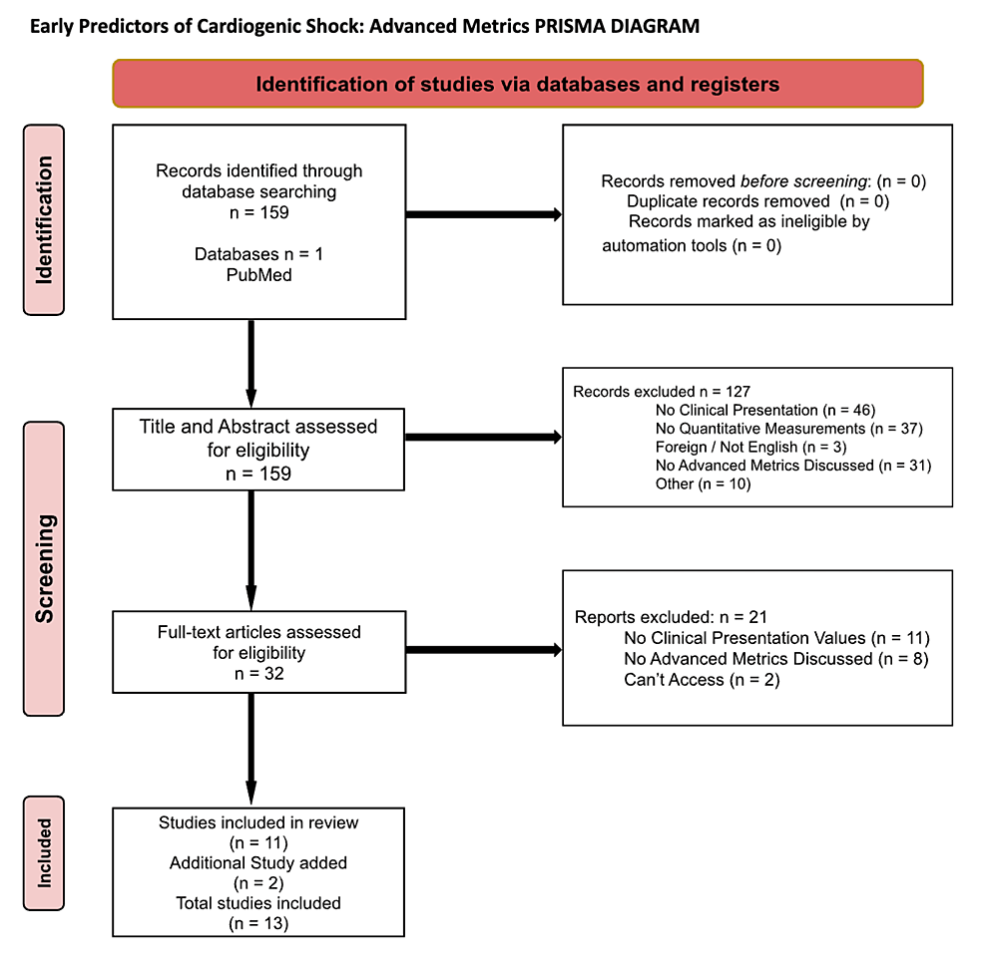
Advanced metrics PRISMA diagram

A total of 159 articles were identified and screened for those that focused on CS, which resulted in the exclusion of 127 articles. The remaining 32 articles discussed clinical presentations and advanced metric findings, such as using pulmonary arterial (PA) catheters. These articles were then screened to identify those that focused on clear clinical presentations as well as quantitative values for laboratory and diagnostic procedural results. This yielded an inclusion of 11 articles. An additional two articles discussing team-based care for CS were also referenced for additional clinical metrics used in diagnosing CS. This process can be visualized in the PRISMA (Figure 1).

**Figure 2:**
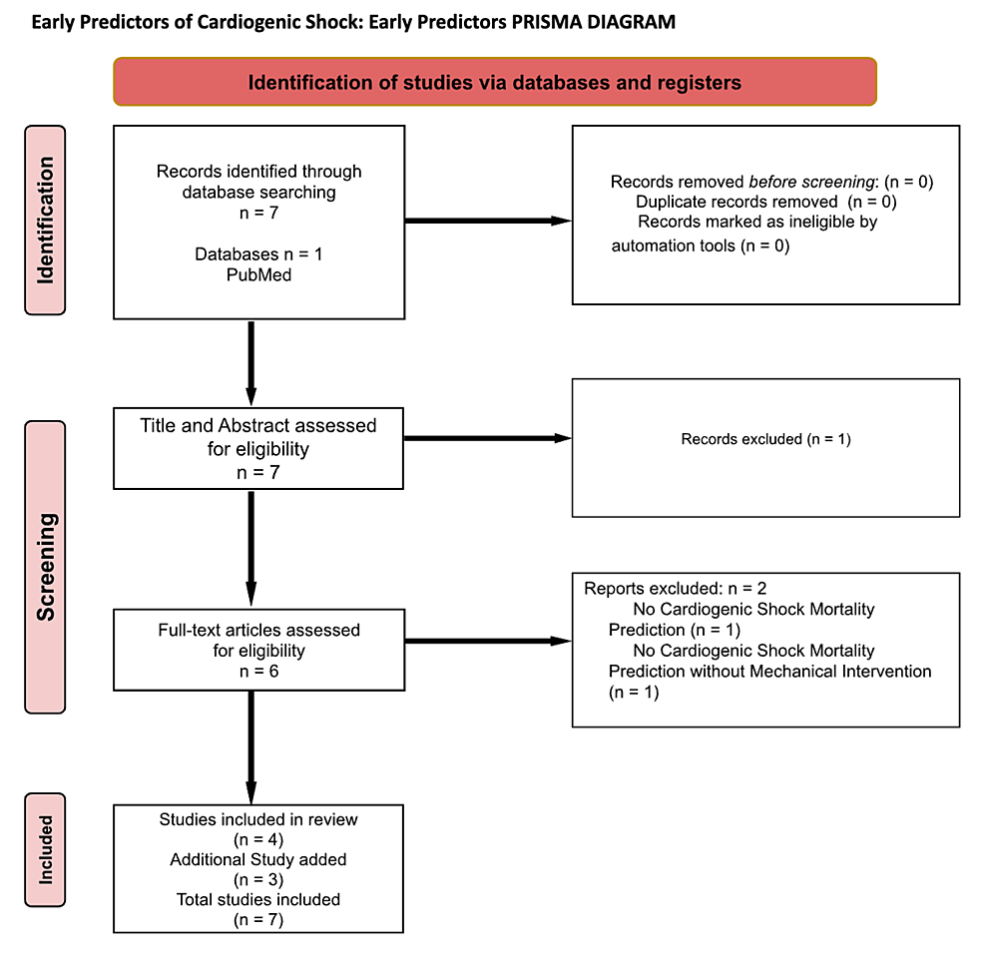
Early predictors PRISMA diagram

A separate search was conducted to assess for early predictors by searching for articles discussing predictors of CS in particular. After identifying six articles that discussed various methods serving as predictors for CS mortality and/or CS development, these articles were individually screened to assess what was being measured. After excluding two studies, one of them being a study that was predicting the short-term mortality in patients with MI complicated by heart failure and the other being a study that was predicting how to wean extracorporeal membrane oxygenation (ECMO) treatment, there were four articles left to be utilized. In addition, two articles were also referenced for additional methods for predicting CS development and mortality, along with the addition of a supplemental article to provide more information on specific topics mentioned. This PRISMA diagram can be utilized for further visualization (Figure 2).

## Review

Clinical presentation/advanced metrics

To address CS, patients must be evaluated clinically, along with advanced metrics, to accurately diagnose and risk stratify the patients to guide management. Individuals exhibiting signs of CS may present with cold and cyanotic extremities, altered mental status (somnolence and confusion), decreased urine output of less than 30 mL per hour (normal urine output is 800-2000 mL per day with an intake of around 2 L of fluids), serum lactate levels greater than 2 mmol/L (normal serum lactate levels are less than 2 mmol/L), and systolic pressure below 90 mmHg for greater than 30 minutes (normal systolic pressure = 120 mmHg) [1]. These are all signs of end-organ hypoperfusion. Patients may also present with pulmonary congestion and laboratory findings of metabolic acidosis (pH less than 7.35) such as an elevation in lactic acid and/or decrease in bicarbonate, due to an imbalance of metabolites and inadequate blood circulation [1]. As the required oxygen delivery gets diminished due to CS, organs and tissue cannot efficiently perform aerobic metabolism, which is the use of oxygen to create energy products, mainly ATP, for our bodies to use in activities. Through the citric acid cycle and oxidative metabolism, a large amount of ATP is created to serve as the body’s primary energy source. When this pathway is compromised, the body switches to anaerobic metabolism, which creates less ATP but in a shorter amount of time. This leads to an available source of ATP in times of low oxygen intake. The increase in anaerobic metabolism creates lactic acid as a by-product, ultimately leading to metabolic acidosis. The most common cardiac site of heart failure is the left ventricle; when it is unable to adequately pump blood, the blood starts to get backed up into the left atrium, which in turn, backs up into the pulmonary veins and lungs, leading to pulmonary congestion.

PA catheters (previously known as a Swan-Ganz catheter) help monitor hemodynamic status and changes, specifically the pressure in the right ventricle and pulmonary arteries along with an estimation of the filling pressure in the left atrium, in intensive care unit (ICU) patients with CS [22]. Hemodynamic monitoring, including measurements using a PA catheter, can also provide signs of CS. These signs and measurements include requiring catecholamine or vasopressor support to maintain an SBP above 90 mmHg and a cardiac power output of around 0.6 W (normal cardiac power output is 1 W at rest). In addition, having a pulmonary capillary wedge pressure (PCWP) greater than 15 mmHg (normal PCWP is 4-12 mmHg), a pulmonary artery pulsatile index (PAPi) of less than 1.0 (normal PAPi is greater than 1.0), and a cardiac index less than 2.2 L per minute per square meter (normal cardiac index ranges from 2.5-4.2 L per minute per square meter) with inotropes or vasopressors, indicate a failing myocardium and suspected CS [23]. Furthermore, differences in CS presentation have been identified between women and their male counterparts. As mentioned above, women are at higher risk of getting CS, so understanding the trends within different genders, along with ethnicities, is vital to effectively addressing and managing CS. Using patient data, including age, smoking activity, comorbidities, and initial LVEF, from a tertiary hospital regarding patients from 2013 to 2019, it was concluded that women who presented with CS were significantly older than the men who presented with CS, with the ages being 76 and 70.8, respectively [24]. In the female population, there was a lower proportion of smokers, a numerically smaller number of patients with chronic obstructive pulmonary disorder as a comorbidity, and a higher prevalence of hypertension when compared to men. The initial LVEF at the time they presented with CS was also significantly higher among women as compared with men, with the ejection fractions being 33.57% and 28.42%, respectively. Both female and male populations had lactate levels above 5 mmol/L, whereas normal lactate levels should be 0.5-2.2 mmol/L [24].

Early predictors of CS 

**Figure 3:**
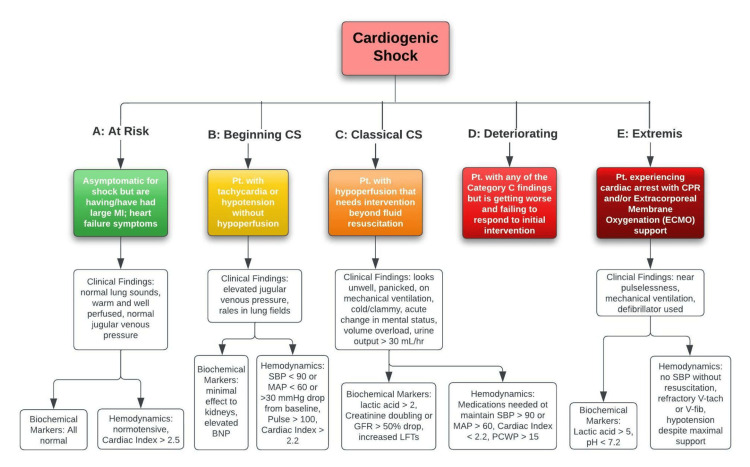
CS Stage Classification created by the SCAI Credit: Original Figure © Nisarg Shah Reference: [25] BNP: Brain natriuretic peptide; GFR: Glomerular filtration rate; V-tach: Ventricular tachycardia; V-fib: Ventricular fibrillation; SCAI: Society for Cardiovascular Angiography and Interventions; CS: Cardiogenic shock; MI: Myocardial infarctions; SBP: Systolic blood pressure; MAP: Mean arterial pressure; PCWP: Pulmonary capillary wedge pressure

In 2019, the Society for Cardiovascular Angiography and Interventions (SCAI) developed a tool to help researchers and clinicians identify the stages of CS. This tool categorizes CS into five stages labeled A “at risk” through E “extremis” (Figure 3). Stage A consists of “at risk” patients who are asymptomatic for shock but have a history of MI or current heart failure symptoms. Stage B consists of patients who are at the “beginning CS” stage with a symptomatic presentation of tachycardia without a decrease in hypoperfusion. Stage C is termed “classical CS” which involves patients who have decreased perfusion that require a more complex intervention than basic fluid retention. Stage D is the “deteriorating” stage where patients present with any of the Stage C findings but are progressively getting worse with no positive response to interventions. Stage E is termed the “extremis” stage, where patients are experiencing cardiac arrest despite cardiopulmonary resuscitation and/or ECMO support. Stages A and B are the most clinically relevant in outpatient settings as these are the findings that would most often present to primary care clinics. This classification tool was created to decrease the ambiguity in defining CS in patients based on clinical presentation so that proper communication can be conducted when referring to CS.

The Observatoire Régional Breton sur l’Infarctus (ORBI) score has also been used to predict the development of CS in hospitalized patients, but this can only be applied to patients who have suffered a STEMI who have survived and undergone a primary angioplasty [26]. The vasoactive inotropic score (VIS), which measures the amount of inotropic and vasopressor therapy needed to sustain adequate blood flow in a patient suffering from CS, is also directly correlated with the mortality of patients with CS when using medical therapy alone [27].

In a study published in 2021 by Yu et al., low levels of serum ionized and total calcium on admission were found to be independent predictors of all-cause mortality in patients who developed CS related to nonischemic cardiac conditions [28]. Using the lowest quartile for ionized calcium, there was a correlation established with low ionized calcium and an increase in all-cause mortality. In a different study that explored the prognostic value of elevated liver function tests (LFTs) in CS patients, it was found that an increase of 20% or more in LFT above normal ranges pointed towards higher mortality in CS if the increase occurred in the first 24 hours of onset [29]. The LFT most indicative of CS mortality was alanine transaminase (ALT). In this setting of CS, an increase in ALT indicated hypoperfusion and increased central venous pressure. Other LFTs showed no relationship to CS risk [29]. Serum lactate trends, which is an end-product of anaerobic metabolism as mentioned above, within the first 24 hours also showed to be of prognostic value in determining 30-day mortality according to a study by Scolari et al. [30]. Lactate levels have been used as prognostic markers for other pathologies as well, such as predicting the 30-day mortality risk for sepsis, the clinical prognosis regarding tumor progression through lactate’s immunosuppressive characteristics and the Warburg effect (the production of lactate even in aerobic metabolism due to the high uptake of glucose by tumor cells), and the mortality risk for patients with acute respiratory distress syndrome receiving veno-venous extracorporeal membrane oxygenation (VV-ECMO) [31-33].

Low diastolic blood pressure (DBP) can also be used as a predictor. Meanwhile, a study conducted on DBP had several limitations that prevent it from being used as an accurate predictor as of now, such as the small sample size and the inability to create a statistically significant correlation between low DBP and CS mortality risk [34]. The neutrophil percentage-to-albumin ratio (NPAR), which has been used to predict the mortality for systemic inflammatory conditions, has also been shown to be independently associated with the mortality rate for CS, which can be a way to predict mortality rates in patients with CS by drawing blood; however, this study showed that although there is a correlation, the sensitivity and specificity of NPAR for predicting CS mortality were low given that the area under the receiver operating characteristic (ROC) curve only ranged from 0.66 to 0.69 (area should be greater than 0.8 to be considered acceptable) [35].

Discussion

With CS often being fatal, it is important to investigate methods to track its development and predict outcomes early so that medical professionals can intervene earlier using pharmacologic or mechanical treatments. Since 2012, several studies have been published researching various metrics including, but not limited to, serum ionized and total calcium, ALT, and the NPAR. Several predictors are validated to be used only when certain etiologies are present, such as the ORBI score only being implemented for patients who have suffered a STEMI and have received treatment through primary PCI. Early predictors of CS presented in this review can be categorized by either being a tool/score or a laboratory measurement.

Tools/scores

Scores/tools used as early predictors of CS include the categorization tool created by SCAI, the ORBI score to predict the development of CS in patients who have survived STEMI and have undergone a primary angioplasty, and the VIS to predict the amount of inotropes and vasopressors needed for a patient suffering from CS. Based on multiple studies utilizing the tool developed by SCAI, updates were made in 2021 that refined the stages to include patients presenting with CS with or without ACS, patients who were in a cardiac intensive care unit (CICU), and patients who presented with cardiac arrest in out-of-hospital settings [36]. It should be noted that although the stage classification has proved to be beneficial in communicating patient statuses to guide treatments for CS, it cannot be used as a stand-alone tool to predict mortality risks as mortality risks are impacted by other factors, such as age and electrolytes levels, that are not incorporated into the stage classification. As such, this tool serves as an effective first step in identifying the severity of CS, but further studies and integration of additional risk modifiers are needed to use it as an all-encompassing predictor of CS mortality risk.

The ORBI score was developed by researchers through a cohort of 6838 patients which incorporated 11 variables independently associated with the development of CS. These variables include age greater than 70 years old, previous stroke or transient ischemic attack, cardiorespiratory arrest on admission, previous STEMI, delay from initial medical contact to percutaneous transluminal coronary angioplasty greater than 90 minutes, Killip-Kimball classification (used for predicting the risk of in-hospital death and potential benefit of specific care in patients with ACS) on admission, heart rate on admission greater than 90 beats per minute, SBP less than 125 mmHg and pulse pressure 45 mmHg, capillary blood glucose greater than 180 mg/dL, lesion of the left main coronary artery, and the thrombolysis in MI flow less than three post PCI. Ultimately, 424 patients were registered for this study that met the criteria. The ORBI score, with an area under the curve-receiver operating characteristic of 0.80 (95% CI 0.73-0.88), had a lower discriminatory capacity with the new population as opposed to the population with which the score was developed. This difference can be attributed to a difference in sample size and risk profile; the patients in this study had a risk profile that aligned with more risk factors [26]. The limited sample size prohibits this study from being generalized to a large population, but it serves as an effective tool to recognize patients at risk for developing CS who have undergone a PCI after STEMI.

The VIS study was a retrospective study conducted at the Samsung Medical Center from 2012 to 2015. The definition of CS that the researchers used was requiring inotrope or vasopressor support to maintain SBP above 90 mmHg and having serum lactate levels above 2.0 mmol/L. Ultimately, 493 patients were eligible to participate in the study, who were then divided into five groups based on VIS: 1 to 10, 11 to 20, 21 to 38, 38 to 85, and greater than 85. The VIS was calculated as follows: dopamine dose (µg/kg/minute) + dobutamine dose (µg/kg/minute) + 100 x epinephrine dose (µg/kg/minute) + 100 x norepinephrine dose (µg/kg/minute) + 10 x milrinone dose (µg/kg/minute) + 10000 x vasopressin dose (unit/kg/minute). Findings from this study included VIS being associated with in-hospital and cardiac ICU (CICU) mortalities in patients with CS needing critical care, VIS serving as a significant prognostic factor for in-hospital mortalities, VIS being able to predict in-hospital and CICU mortalities better than serum lactate levels and the Acute Physiology and Chronic Health Evaluation II score, and VIS showing a weak correlation with probability of in-hospital mortality in patients treated with ECMO with a VIS of 130 or higher [27]. At VIS less than 85, ECMO use correlated with higher mortality, likely due to ECMO-related systemic inflammatory response syndrome (SIRS) [27]. Therefore, although randomized controlled studies must be conducted to determine a strong correlation between VIS and ECMO use, incorporating the VIS into management algorithms allows clinicians to have a parameter to guide ECMO use. This study also focused on the medications used to calculate the VIS; future studies would have to consider other medications that are being used in the management of CS. Furthermore, the APACHE II score was used as the standard measurement to determine the potential for the VIS to predict mortality; this was due to the absence of a standard risk-stratification system for CS, further adding to the necessity for the establishment of a gold-standard CS predictor tool.

Laboratory measurements

Laboratory measurements used as early predictors of CS include serum ionized and total calcium, ALT, serum lactate, DBP, and the NPAR. Serum calcium on admission was used to correlate with mortality rates in critically ill patients with CS. This was a single-center retrospective study that had 921 patients evaluated. Using the lowest serum ionized calcium quartile with levels being less than 1.04 mmol/L (normal serum ionized calcium levels are 1.2-1.4 mmol/L), results showed an independent association with an increased risk in the 30-day mortality (p = 0.049), 90-day mortality (p = 0.030), and 365-day mortality (p = 0.046) that were all statistically significant [28]. Low serum calcium has also been associated with a poor prognosis for diseases such as coronary arterial disease, heart failure, acute kidney injury, and chronic kidney disease [28]. Considering this study was conducted retrospectively at a single center, future studies will need to be large prospective studies to confidently apply the findings to other centers and populations. Moreover, considering that movements like patients repeatedly pumping their fists prior to a blood draw affect local pH, which affects calcium binding to protein, a standard collection protocol must be established and implemented to measure consistent serum calcium readings.

As CS can affect other organs in addition to the heart, abnormal LFTs are often seen in heart disease. Dr. Jantti, Tarvasmaki, Harjola, et al. conducted a prospective, observational, multicenter, and multinational study on CS where LFTs were measured serially at 12-hour intervals to potentially discover a predictive power in the LFTs in predicting mortality in patients suffering from CS [29]. CS was defined in this study as an SBP less than 90 mmHg after a proper fluid challenge for 30 minutes or a need for vasopressor therapy to maintain SBP above 90 mmHg, as well as patients exhibiting signs of hypoperfusion, which includes altered mental status, cold peripheries, oliguria of less than 0.5 ml/kg/hour for six hours, or blood lactate greater than 2 mmol/L. Substances that were analyzed included C-reactive protein, creatinine, high-sensitivity troponin T, ALT, alkaline phosphatase, gamma-glutamyl transferase (GGT), and total bilirubin. A total of 178 patients were included in this study. The results of this study showed that an increase in ALT levels by at least 20% within the first 24 hours was independently associated with an increase in mortality of patients with CS. Independent predictors of early increases in ALT included low LVEF, low mean arterial pressure, low C-reactive protein, and low estimated glomerular filtration rate [29]. Furthermore, early increases of other LFTs such as GGT, bilirubin, and alkaline phosphatase showed no statistically significant association with the 90-day mortality [29]. A limitation of this study is that the 20% cutoff was established from prior studies regarding heart failure, so the association between a 20% increase in LFTs and cardiac disease needs to be further established through additional studies. Despite this limitation, this study shows the potential of monitoring ALT changes in patients with CS to predict the chances of mortality and should be a key laboratory marker in CS prognostication.

Serum lactate has been historically used to monitor several pathologies [31-33]. Scolari et al. conducted a retrospective analysis of a group of CS patients treated with Impella CP or veno-arterial ECMO (VA-ECMO) at four tertiary centers in Brazil [30]. Forty-three patients were included in this analysis. To be included in the study, hemodynamic instability was defined as SBP less than 90 mmHg despite vasopressor and inotrope administration, signs of end-organ failure such as clammy skin, capillary refill time greater than three seconds, lactate level greater than 4 mmol/L, and urine output less than 0.5 mL/kg/hour, and low cardiac output [30]. All lactate levels were measured in arterial blood gas, and levels were measured at one hour, six hours, 12 hours, and 24 hours post-device implantation. Results from this study showed that lactate levels at all time points and lactate clearance after six hours were associated with 30-day mortality. Additionally, levels of serum lactate at six hours, 12 hours, and 24 hours as well as lactate clearance after 24 hours were able to differentiate between survivors and nonsurvivors [30]. Lactate levels and clearance both showed an increase in area under the ROC curve (AUC) over time with the greatest AUC occurring at 24 hours; however, lactate level AUCs were higher at every time point when compared to lactate clearance AUCs. This indicates that lactate levels are a better prognostic indicator than lactate clearance. A key observation to note is that patients who were unable to clear baseline lactate levels after 24 hours of mechanical circulatory support treatment yielded a 100% mortality [30]. A limitation of this study is the small sample size used, which makes it difficult to extrapolate the findings from this study and apply them to other populations. Protocols at the four centers may have differed in regard to fluid and vasopressor management, which may have caused lactate levels to vary. This study is the first study to explore lactate kinetics in patients with VA-ECMO and Impella CP utilization to predict mortality in patients with CS. It has shown that serum lactate monitoring post-VA-ECMO or Impella CP utilization can provide valuable prognostic information, especially serum lactate levels 24 hours post-implantation. Therefore, lactate levels show potential and should be utilized to guide prognosis and ultimately management of CS.

Using a foundational metric such as DBP has also been shown to predict CS 28-day mortality; this is significant because it can easily be incorporated into any algorithm for CS monitoring and prognostication. Axler conducted a retrospective study in the ICU, which included 71 patients with various etiologies of CS [34]. In this study, DBP values from the first hour, second hour, third hour, fourth hour, and the mean of the lowest diastolic arterial pressure (DAP) during the 24 hours were all averaged to report a single value. The authors believed a single value at any time point had many variable factors contributing to it, so a mean of the values over the course of 24 hours provided a more representative value of the patient’s condition. A key observation made during this study was that patients with CS were dying due to a SIRS which was causing vasodilation consistent with the low DBP. This vasodilation conflicting with the vasoconstriction of heart failure seems to be the mechanism behind the eventual demise of the patient [34]. This study also showed that DAP as well as DBP were consistently low in the nonsurvivor group as compared to the survivor group. The minimum DAP was also shown to be independently associated with the 28-day mortality in patients suffering from CS. A limitation to note, as mentioned above, is the small cohort size of this study as well as the retrospective and singular center nature of this study, which calls for a prospective, larger cohort study to be conducted before the observations made here can be validated. Additionally, an ROC curve was not possible in this scenario due to the limitation of the variables included in this study, which calls for an addition of variables in future larger studies. In a setting where risk assessment and treatment efficiency are significant, an uncomplicated measurement like DBP can guide a clinician.

A comprehensive metabolic panel (CMP) and a complete blood count (CBC) with differential are common lab tests that are ordered in hospital settings to monitor how patients are doing. The neutrophil percentage is included in the CBC with differential, and the albumin level is included in either the LFT or CMP. In patients with CS, gaining a prognosis from these two lab tests by using NPAR allows physicians to tailor their care appropriately early in management, which can potentially lead to better outcomes for patients. As CS can be caused by SIRS, inflammation can be monitored by looking at neutrophil counts. In the study conducted by Yu and colleagues, 891 CS patients were selected from the Medical Information Mart for Intensive Care-III database [35]. This study measured all-cause in-hospital mortality primarily, as well as all-cause 30-day and 365-day mortality. Results showed a nonlinear relationship between NPAR and in-hospital and 30-day mortality (all p-values < 0.001). Based on the cut-off values made by X-tile analysis, NPARs were divided into three groups: group 1 (NPAR < 25.3), group 2 (25.3 ≤ NPAR < 34.8), and group 3 (34.8 ≤ NPAR). Cox analysis showed that a higher NPAR was independently associated with an increased risk of in-hospital mortality (group 3 versus group 1: hazard ratio [HR] = 2.60, 95% confidence interval [CI] 1.72-3.92, p < 0.001), 30-day mortality (group 3 versus group 1: HR = 2.42, 95% CI 1.65-3.54, p < 0.001), and 365-day mortality (group 3 versus group 1: HR = 6.80, 95% CI 4.10-11.26, p < 0.001) [35]. AUCs for in-hospital mortality for the NPAR, neutrophil percentage, and albumin were 0.69 (95% CI 0.66-0.73), 0.56 (95% CI 0.53-0.61), and 0.57 (95% CI 0.53-0.61), respectively [35]. This study also showed that admission NPAR was an independent predictor of clinical outcomes in CS patients as well. Additionally, through measuring AUCs, NPAR was a better predictor of outcomes than albumin or neutrophil percentage alone [35]. Limitations of this study included its retrospective, singular center design, which gives it a risk for selection bias and should be conducted on a larger scale with additional variables to observe a stronger correlation. There are also known and unknown factors that may confound the results, which adds to the reason for conducting a larger study that considers additional variables. Additionally, the ROC for NPAR was low; therefore, incorporating the NPAR into a multivariable scoring system will provide more prognostic value than using the NPAR as an individual metric [35].

Future studies incorporating additional databases and covering additional languages should be conducted to gain a more encompassing review. Further studies also need to be conducted to determine which optimal metric should be used to assess the broad spectrum of CS while also providing the most accurate prognostication. The prognostic metrics must also be validated to establish sensitivities and specificities. In addition, multiple databases should be utilized to get a more thorough examination of the various modalities that are in existence today that can help predict CS mortality and severity.

## Conclusions

Approximately six million people die from sudden cardiac death globally every year, with 300,000 to 400,000 of them happening in the United States In addition, 40,000 to 50,000 individuals within the United States are affected by CS following an MI, with 40% of individuals dying within 30 days. Challenges to treating CS include the lack of an established algorithm that can be transitively used with different etiologies of CS and the inability to effectively anticipate or prepare for the onset of CS in a patient. The aim of this review is to illuminate several predictors of CS that have been studied over the past 10 years that show promise for predicting the severity and mortality of CS. The findings from this review prove to be beneficial as they address the high mortality rate of CS. Examining these predictors will allow earlier intervention in the development of CS and potentially help lower the mortality rate of CS. Through conducting this review, it is believed that the eventual creation of a scoring system that incorporates multiple metrics discussed in this review, such as ALT, serum lactate levels, NPAR, and DBP, can provide the most accurate prognosis of CS, which can lead to more targeted interventions. Incorporating new technologies and methods to combat a global illness pushes the field of cardiology forward by creating the opportunity to intervene earlier in the disease progression of CS than ever before. Clinical practice may one day be dictated by a thorough algorithm that integrates a multitude of these predictive factors.
